# Preservation of Carious Teeth by Filling

**Published:** 1863-12

**Authors:** Enoch C. Rolfe


Preservation of Carious Teeth by Filling—*By Enoch C. Rolfe*, M. D.—So far I have spoken of teeth with dead or exposed nerves only, without stopping to consider the other conditions of the tooth. The nerve may be exposed, however, while the decay is quite limited, and situated in the articulating surface, where it can be readily filled with common dentists' foil. The treatment of such a case is as plainly indicated, as that for abscess or carbuncle, and yet there are dentists and physicians who insist that extraction is the proper remedy for all teeth with dead or exposed nerves.

The second form of diseased teeth, is where, besides having an exposed nerve, there is loss of a part of the cutting surface or crown of the tooth.

Under most circumstances it may be better to remove such teeth; for instance, where we find such teeth, nearly all the other teeth may be diseased, the gums spongy and otherwise diseased, the teeth covered with tartar, and perhaps alveolar abscess is to be added to the rest. Oftentimes a person may have two, three, or four teeth, that are worth saving, but the others are gone, or too much diseased for cure. Under all the above conditions the best treatment is to remove the whole, and give the patient an artificial set. But it is a very common thing to see patients with one, two or more front teeth badly decayed, while most of the other teeth, and perhaps all, are good, and the mouth and gums in a healthy condition. If these two or three diseased ones can be restored to health and shape, the patient may avoid the necessity for wearing artificial teeth for years, perhaps for life.

Where only one or two front teeth are decayed, if the fangs are healthy, they may be cut off and artificial ones pivoted upon the roots; but pivot teeth are filthy nuisances in the mouth under the most favorable circumstances. Any person who has worn one, would be willing to pay any reasonable sum to have his old tooth back again, if it could be filled and made clean and healthy. One or two teeth may be attached to a plate, made fast to the other teeth by clasps or springs, or the plate may be retained in place by atmospheic pressure. Either of these is far preferable to pivots, but clasps soon destroy the teeth they emprace, and atmospheric pressure is disagreeable, and a constant drag upon the roof of the mouth.

By plugging from one to half a dozen, oftentimes all these artificial appliances may be avoided, and perhaps severe alveolar abscess or neuralgia cured.

So long as the only form of gold prepared for filling teeth was that known as "dentists' foil," it was hardly possible to restore a tooth that had any considerable portion of its crown or cutting surface or original shape destroyed, although long practice had enabled some operators to perform surprising cures with this material. Mr. Alfred J. Watts, of Utica, N. Y., has patented a method of preparing gold, which renders it so soft that it can be packed layer upon layer, by pressure, until it becomes a solid mass, equal in density to coin. With this gold, a tooth with a portion of its crown broken away, may be built out to its original shape, if a firm foundation can be obtained to commence upon. His method of preparing is simple. He first dissolves pure gold in itro muriatic acid. It is then precipitated with proto-sulphate of iron; this precipitate is washed with hydrochloric acid to remove any peroxide of iron or other impurities, that will thus be washed away. This precipitate is then amalgamated with from four to twelve times its weight of mercury. The mercury is then dissolved out with nitric acid, leaving the gold in the form of a brittle non-coherent mass. It is then annealed, or slowly raised to a cherry-red heat, and it is ready for use.

With gold thus prepared, by careful manipulation, an old broken tooth may not only be restored to its original shape, but its weak parts strengthened, and bound together.

Case I.—The wife of one of the members of this society, while having an operation performed upon another tooth, called my attention to one of the central incisors, saying she supposed nothing could be done for that, but wait for it to break and then have its place supplied by an artificial one. Upon examination, I found that the nerve was dead and had probably been so for a long time; that about one-fourth of the cutting surface upon the lateral side was gone—a little less on the labial aspect, a little more on the palatine, and the tooth much discolored. On cutting into the decay I found that the discoloration could readily be removed. The gums were in a healthy condition; most of the other teeth had been plugged, but were in good condition. I ventured to promise that I could plug it, restore its original shape and nearly restore its color, and make a useful tooth of it, which would probably last as long as her other teeth.

But I also told her that there was a possibility that the tooth would break in the operation, but that if it would break during an operation, it would soon break anyway. She said if she could be sure that it would not break she would not value any money, for she had a perfect horror of artificial teeth, but the idea that it might break frightened her. She left, however, saying that she would hear what the Doctor had to say about it and see me again. She came again in two or three days, saying that her husband advised her to put the case into my hands to do with, just as I thought best. I plugged the root to the apex, restored the crown to its original shape, and left it with nearly as good a color as its mate which was sound. I saw her after three-quarters of a year's use; she expressed herself more than satisfied with the success of the operation; said the tooth seemed everyway as good as the other, and that no money would induce her to have it put in the condition in which I found it.

Case II—Another physician's wife came to me with an under bicusped badly decayed, part of the crown gone, nerve exposed but not dead. This was treated like the first, after killing the nerve. She then informed me she had been to another dentist, who had told her that the tooth was too far gone for any operation but extracting, and rather insisted on removing it at once. She was so unwilling to lose the tooth, that she concluded to seek further advice; her husband sent her to me, and the result is that she now says she would be willing to pay fifty dollars to have one like it filled, rather than lose it.

Case III.—Mrs. S., a lady from Maine, consulted me about her upper front teeth. Except these four, her teeth were very good. These had been decayed for many years, and were very bad. The centrals were decayed through from side to side, the nerves dead, and the nerve-cavities so decayed that they were tunnel-shaped; one-third of the cutting surface upon each side was gone. The laterals were about as badly decayed upon the central side, but sound upon the cuspid portion; nerve-cavities not so badly decayed. With one she had had alveolar abscess frequently; these teeth were all badly discolored. This to appearance was a most unpromising case—at the time, the most unpromising one I had ever seen. The lady was a resolute woman, determined from principle never to wear artificial teeth, but to keep her own as long as possible. She was therefore ready to have anything done that I dared undertake. The resolution of the patient, healthy gums and naturally strong teeth, were in my favor. I commenced by plugging the fangs of the centrals at the first sitting. One inflamed and abscess formed. A less resolute woman would have given up; she poulticed her face, and in four days returned as resolute as ever. At the next sitting I treated the laterals as I had before done the centrals. At four sittings of about two hours each, I succeeded in safely plugging the six cavities, completely restoring the shape and size of the teeth and very much improving their color. Into these six cavities I had condensed five pennyweights of gold. I had made it a condition before operating that she should stay in town after the operation until I should be satisfied that all would do well. At the end of a week there were no signs of inflammation or soreness, and I allowed her to go home. I saw her nearly a year after the operation, and she told me her teeth had not given her the slightest trouble or uneasiness; that they felt so much like her teeth of former days, that she feared she might use them too carelessly, and some day undertake to crack nuts or some other hard substance, and break them. She, too, said no money could buy the gold from these teeth unless more could be put in.

Case IV.—The master of one of the South-End Schools had a lateral incisor that had been filled more than a quarter of a century. Until within the last year it had not given him the slightest trouble, when, all at once, the filling fell out. To his astonishment, he found that decay had commenced upon the other side of the tooth, extending entirely through it until it had loosened the filling which was upon the central side. When I saw the tooth the cavity extended from side to side, one-quarter of the front point next the central was gone, and all the articular surface except the enamel. Tho tooth looked very much as if a saw had been run across it, which was just thick enough to take all but the front enamel, but as it went up and the tooth became thicker, it left the enamel on the palatine portion. The decay extended upward to about a level with the alveolus, but the nerve did not seem to be involved, probably the nerve was dead and the cavity filled with ossific matter. The question with the patient was, whether it was best to cut off the tooth and pivot one upon its root, or extract it and set a tooth upon a plate. The latter could be easily done, as he already wore the centrals on plate. I told him there was no need of doing either, that I could make that tooth more serviceable than any artificial tooth could be, but it would proclaim its golden character. To him this was no objectien. I filled it, and he seemed delighted with its result. If it lasts as long as the first he promises to be satisfied, and I think I shall be. He said it was worth fifty dollars to him, and he was willing to pay me that for the operation. I concluded to take a more moderate fee, telling him that at the end of the twenty-five years, if the filling was good, I would take the balance.

[Dr. Rolfe also exhibited a patient in whom he had restored to its original form and dimensions about a quarter of the substance of an upper incisor tooth, extending from the gum to the extremity on its outer edge.]—*Bost. Med. & Sur. Jour.*

The physicians of Boston are again moving in behalf of a Government appropriation to Dr. Morton, the alleged discoverer of "practical anæsthesia." A circular has been issued calling upon medical men to influence the representatives and senators in Congress with whom they may have acquaintance. It is signed by Dr. James Jackson, President of the Morton Testimonial Association, and by Dr. John Ware, Chairman of the Executive Committee.—*Am. Med. Times.*

